# Sudden rupture of recurrent rectal prolapse complicated by small intestine evisceration from anus: a case study

**DOI:** 10.3389/fmed.2025.1581332

**Published:** 2025-05-27

**Authors:** Zhesen Tian, Xiaopeng Ma, Mingda Li, Hongxun Ruan, Guojian Zhang, Yalei Zhao

**Affiliations:** ^1^Department of Radiotherapy, The Second Hospital of Hebei Medical University, Shijiazhuang, Hebei, China; ^2^Department of Anus and Intestine Surgery, The Second Hospital of Hebei Medical University, Shijiazhuang, Hebei, China; ^3^School of Basic Medical Sciences, Hebei Medical University, Shijiazhuang, Hebei, China

**Keywords:** rectal prolapse, small intestine evisceration, Hartmann operation, emergency treatment, case report

## Abstract

Evisceration of the small intestine from the anus is a rare complication following rectal injury. In patients with recurrent rectal prolapse, delayed surgical intervention can lead to rectal wall thinning due to chronic ischemia, increasing the risk of rectal rupture and subsequent intrusion of the small bowel followed by antegrade intussusception. Since Bodie first described this condition in 1827, fewer than one hundred cases have been reported globally. This case involved a 78-year-old male with a 60-year history of untreated rectal prolapse. After a fall, his abdominal pressure increased abruptly, causing rectal rupture and intussusception of the small intestine through the anus. An emergency Hartmann procedure was performed, and the patient recovered well. This case underscores the need for early surgical intervention in chronic rectal prolapse to prevent severe complications. It also highlights the unique mechanism of rectal injury under external force, providing valuable insights for managing similar complex cases.

## 1 Introduction

Rectal prolapse (RP) refers to the partial or full-thickness downward displacement of the rectal wall, with protrusion partially or entirely beyond the anal orifice (1). This condition predominantly affects women and older adults, with a sixfold higher incidence in women compared to men. Elderly individuals, particularly those with chronic constipation and pelvic floor dysfunction, exhibit elevated susceptibility to RP and are prone to severe complications that significantly impact quality of life (2). Clinical manifestations include fecal incontinence, obstructive defecation, incomplete rectal emptying, and pelvic pain (2, 3). Complete rectal prolapse may precipitate rare but life-threatening sequelae, such as gangrenous necrosis or perforation (4).

RP is often associated with anatomical and functional abnormalities of the pelvic floor, such as elongation of the sigmoid colon, deepening of the rectovesical or rectouterine pouches, relaxation of the levator ani muscle, and weakness of the pelvic floor fascia (1). Furthermore, rectal prolapse is also related to one’s own nutritional status, age and the level of intestinal inflammation (5). Surgery is the preferred treatment for RP. There are many surgical methods for treating RP, which can be classified into transabdominal and transperineal approaches based on the surgical approach (6, 7). If patients do not receive timely surgical treatment, they may develop chronic and recurrent rectal prolapse, which can lead to a series of complications, such as fecal incontinence, necrotizing intussusception, intestinal mucosal ischemia, ulcers, perforation, and even strangulated intestinal necrosis (8). It is worth noting that traumatic external forces (such as falls or sudden increases in abdominal pressure) can further increase the risk of injury to the prolapsed intestinal segment, leading to intestinal perforation, abdominal infection, and other critical conditions (9, 10).

Reports of small intestine evisceration through the rectum secondary to rupture caused by recurrent rectal prolapse are exceedingly rare. This case involves a 78-year-old male patient who had not undergone surgical intervention for recurrent rectal prolapse presenting with transanal evisceration of the small bowel via the ruptured rectum after a fall. By analyzing its pathogenesis, treatment process, and surgical decision-making, it provides valuable insights for managing similar complex cases and prompts reflection on family care for elderly patients.

## 2 Case report

**FIGURE 1:**
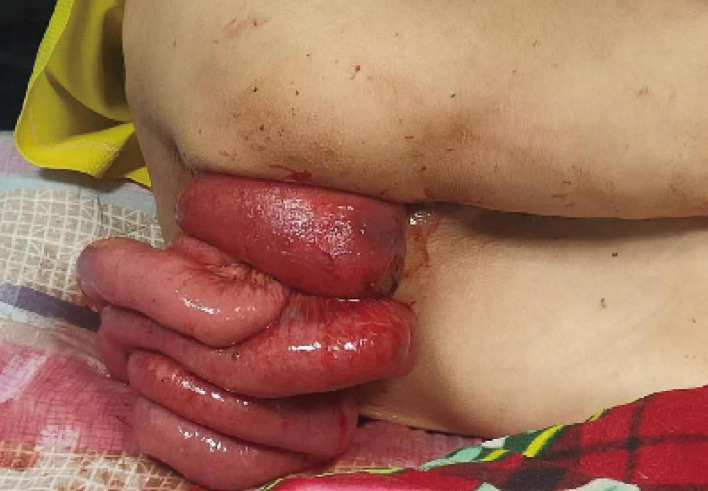
Transanal evisceration of small intestine during admission.

On January 21st, 2025, a 78-year-old male patient presented to the emergency department of our hospital with prolapse of the small intestine and rectum from the anus accompanied by bleeding for 3 h. Three hours ago, the patient experienced RP during defecation. While attempting manual reduction, he accidentally fell and, upon standing, experienced abdominal pain, abdominal distension, and nausea with vomiting. Subsequently, the small intestine and rectum prolapsed from the anus with a small amount of bleeding (Figure 1).

The patient has a 60-year history of RP without surgical intervention and often manually reduces the rectum. There has never been a previous instance of small intestine prolapse from the anus. In 2009, he suffered a car accident resulting in intracranial hemorrhage and underwent craniotomy, after which he developed left lower limb motor dysfunction. In 2018, he had a cerebral infarction and received conservative treatment. In June 2024, he suffered a lumbar vertebra fracture from lifting heavy objects and underwent bone cement augmentation. In October 2024, he had another cerebral infarction and continued to receive conservative treatment.

### 2.1 Investigations

After admission, the vital signs of the patient were relatively stable: body temperature 36.7°, pulse 107 beats/min, respiration 21 breaths/min, blood pressure 123/85 mmHg. Abdominal examination revealed positive signs of peritoneal irritation (abdominal muscle tension, tenderness, and rebound tenderness), and weakened bowel sounds.

**FIGURE 2:**
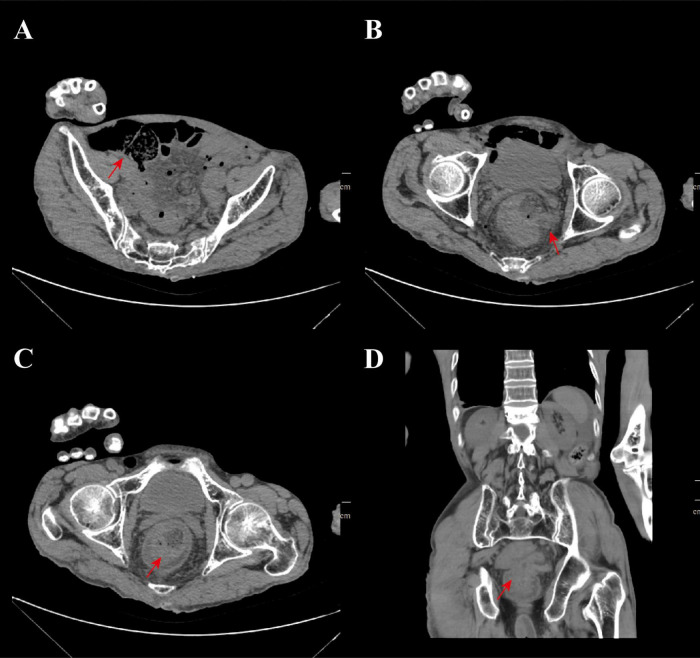
CT imaging findings. **(A)** The CT imaging manifestations of small intestine dilation and inflation, the red arrow indicates the dilation of the small intestine; **(B)** imaging manifestations of small intestine prolapse after rectal rupture and the red arrow indicates the site of rectal rupture; **(C)** the imaging shows that there is intussusception and rectal prolapse and the red arrow indicates the lesion site; **(D)** the coronal plane imaging manifestations of the patient, the red arrow indicates the location of recurrent rectal prolapse and intussusception.

Due to rectal prolapse, the location of the rupture can be reached by digital rectal examination. We disinfected the small intestine with diluted iodophor solution and then sent it back into the abdominal cavity through the rupture. After returning the small intestine and rectum to their normal positions in the emergency department, imaging and laboratory tests were performed to assess the patient’s general condition. Computed tomography (CT) showed multiple free gas in the abdominal cavity, with the small intestine dilated and filled with gas (Figure 2A); diffuse exudation in the abdominal and pelvic cavities with multiple effusions; thickening of the intestinal wall in the lower segment of the rectum, with intestinal images visible inside, suggesting intussusception (Figures 2B, C); the lower end of the rectum protruded outside the anus, suggesting rectal prolapse (Figures 2C, D).

### 2.2 Treatment

**FIGURE 3:**
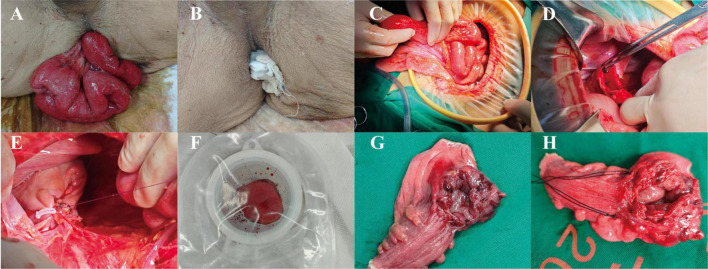
The surgical process of the patient and the gross pathological specimen. **(A)** The condition around the anus of the patient when in the lithotomy position; **(B)** before the operation, the patient’s anus was blocked by gauze; **(C)** The condition of small intestine and abdominal cavity during the operation; **(D)** the location of the rectal rupture; **(E)** Hartmann procedure for distal colon occlusion; **(F)** proximal colostomy formation in Hartmann’s procedure; **(G, H)** Postoperative gross pathological specimen.

The emergency surgery was conducted by colorectal surgeons. Under general anesthesia, the patient was positioned in lithotomy for manual reduction of the small intestine. Examination of the anus and surrounding areas did not reveal any injuries or deformities. The anus was temporarily sealed intraoperatively with gauze (Figures 3A, B). Intraoperative laparotomy revealed no fecal or purulent contamination within the peritoneal cavity (Figure 3C). However, due to the bleeding after rupture and the repeated repositioning of the small intestine, infection occurred, leading to symptoms related to peritonitis. A 2-cm rectal perforation was identified on the anterior wall of the upper rectum, approximately 6 cm above the peritoneal reflection (Figure 3D). Given the patient’s advanced age, poor nutritional status, and concurrent localized peritonitis, resection and anastomosis of the damaged rectal tube is a very risky procedure. Consequently, the decision was made to perform a Hartmann procedure, which involved resection of the perforated rectal segment and creation of a colostomy (Figures 3E, F).

### 2.3 Outcome and follow-up

**FIGURE 4:**
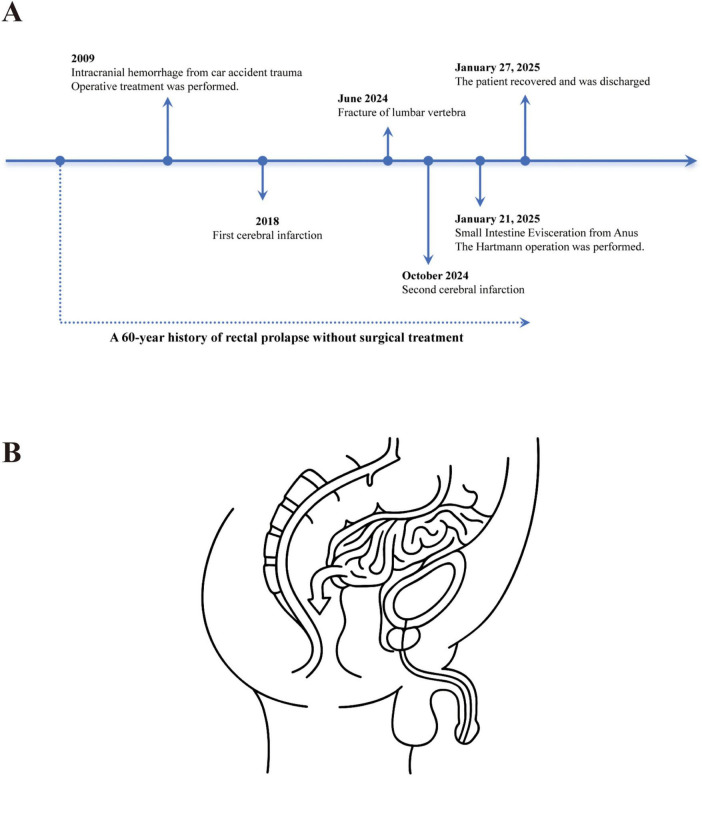
**(A)** A timeline of the patient’s disease progression. **(B)** The illustration to show the path of evisceration of the small intestine.

The patient underwent resection of the ruptured rectal segment approximately 8 cm long, with a breakage point visible about 2.5 cm away from the residual end on one side, measuring about 2.2 * 1 cm (Figure 3G). The gross pathological examination after the operation showed that the intestinal wall around the rupture site was very weak (Figure 3H). Under the microscope, acute and chronic inflammatory reactions of the intestinal mucosa could be seen at the breakage point, as well as full-thickness bleeding of the intestinal wall, with some mucosa detaching and necrotizing accompanied by acute inflammatory exudation, which was consistent with the pathological manifestations of intestinal rupture. The patient recovered normally after the operation and had no other complications. Seven days later, he was discharged smoothly. Whether to undergo colostomy reversal surgery will be evaluated based on the patient’s physical condition. A timeline of the patient’s disease progression is shown in Figure 4A. Figure 4B shows the path of evisceration of the small intestine.

From the perspective of the patient and his family, the emergency surgery was very satisfactory. Besides, in the home care, for patients with rectal prolapse, early surgical intervention is necessary.

## 3 Discussion

Rectal prolapse encompasses various types, including complete and incomplete prolapse, as well as external and internal prolapse. In cases of incomplete prolapse, also referred to as mucosal prolapse, only the rectal mucosa is displaced downward. Complete prolapse occurs when the entire rectal wall descends. Internal prolapse refers to the descent of the rectal wall within the anorectal cavity, while external RP involves the protrusion of the rectal wall outside the anus (11). Complete rectal prolapse can lead to numerous complications, such as damage to the rectum and surrounding structures, incarceration, perforation, strangulation. Abdulgader et al. reported a case of incarcerated rectal prolapse (12). This study is a special case of rectal prolapse complicated with rupture and perforation.

Transanal intestinal evisceration is a rare complication of chronic RP. Chronic prolapse can lead to ischemia of the anterior rectal wall, and repeated episodes of ischemia over time result in thinning of this region. In such a compromised state, a sudden and significant increase in intra-abdominal pressure can cause perforation at the weakened area of the rectum, allowing the small intestine to protrude through the damaged rectal wall into the anus (10). This condition was first described by Brodie in 1827 (13). Over the subsequent two centuries, approximately 100 cases of transanal small intestinal prolapse following rectosigmoid rupture have been reported globally. Most patients presented with a history of recurrent rectal prolapse and had not undergone timely surgical intervention during the early stages of their condition. Upon presentation, these patients often exhibited urgent and life-threatening conditions, leading to a high mortality rate. Historically, patients with rectal rupture and transanal small intestinal evisceration almost invariably experienced factors that increased intra-abdominal pressure, such as defecation, vomiting, heavy lifting, or manual reduction after prolapse (14). The development of colostomy and Hartmann surgery has significantly improved outcomes; from 1979 to 2021, the mortality rate for patients with rectal rupture and transanal small intestinal prolapse decreased from 63 to 13%. Previous case analyses have demonstrated that patients who only received primary repair of the perforation had a mortality rate as high as 80% (15). Combining rectal tear repair with colostomy reduced the mortality rate to 23% (14).

The evolution of the patient’s condition and the process of diagnosis and treatment in this case involve multiple critical clinical issues. Untreated RP for a long time (60 years) represents a potential risk. The recurrent episodes of RP lead to degeneration of the intestinal wall muscle layer, thereby increasing the risk of perforation. Getting up forcefully after falling down, which caused a sudden increase in intra-abdominal pressure and mechanical traction, directly resulted in rectal rupture and small bowel prolapse, indicating that trauma is an important cause of acute rectal injury. In this case, considering the patient’s advanced age, malnutrition and local peritonitis which might increase the risk of anastomotic leakage, the Hartmann procedure (partial rectal resection with colostomy) was chosen.

The advantages of the Hartmann procedure include complete removal of the source of infection, avoidance of anastomotic complications, and preservation of the possibility for future restoration of intestinal continuity. The primary repair is applicable to patients without peritoneal contamination and with no massive bleeding. It can directly repair the perforation site. However, for patients with chronic rectal prolapse, it is prone to increase the risk of recurrence (10). Postoperative gross pathological findings revealed full-thickness bleeding and mucosal necrosis of the intestinal wall, consistent with acute perforation. The smooth postoperative recovery confirms the effectiveness of the emergency surgical strategy, but whether to perform colostomy reversal will depend on a comprehensive assessment of the patient’s neurological function, nutritional status, and surgical tolerance.

This case suggests that for patients with long-term rectal prolapse, surgical intervention should be carried out as early as possible to prevent the occurrence of serious complications. At the same time, when dealing with such complex cases in the emergency department, an appropriate surgical method should be selected based on the specific condition of the patient. This Supplementary material presents the information list of the CARE case report for this study.

## Data Availability

The original contributions presented in this study are included in this article/Supplementary material, further inquiries can be directed to the corresponding author.
