# Supplementary material for: Sudden rupture of recurrent rectal prolapse complicated by small intestine evisceration from anus: a case study
Source: Front Med (Lausanne). 2025 May 27;12:1581332. doi: 10.3389/fmed.2025.1581332 (PMC12149217; doi:10.3389/fmed.2025.1581332)
Supplement: Supplementary file 1 [file Data_Sheet_1.PDF]

CARE
case report guidelines

CARE Checklist of information to include when writing a case report

(cc) BY-NC-ND

Topic	Item	Checklist item description	Reported on Line
Title	1	The diagnosis or intervention of primary focus followed by the words "case report"	Yes Title
Key Words	2	2 to 5 key words that identify diagnoses or interventions in this case report, including "case report"	Yes Lines 12—13
Abstract (no references)	3a	Introduction: What is unique about this case and what does it add to the scientific literature?	Yes Lines 17—24
	3b	Main symptoms and/or important clinical findings	Yes Lines 19—21
	3c	The main diagnoses, therapeutic interventions, and outcomes	Yes Lines 20—22
	3d	Conclusion—What is the main "take-away" lesson(s) from this case?	Yes Lines 23—24
Introduction	4	One or two paragraphs summarizing why this case is unique (**may include references**)	Yes Lines 47—65
Patient Information	5a	De-identified patient specific information.	Yes,we de-identified the patient information.
	5b	Primary concerns and symptoms of the patient.	Yes Lines 67—72
	5c	Medical, family, and psycho-social history including relevant genetic information	Yes Lines 73—79
	5d	Relevant past interventions with outcomes	Yes Lines 73—79
Clinical Findings	6	Describe significant physical examination (PE) and important clinical findings.	Yes Lines 81—84
Timeline	7	Historical and current information from this episode of care organized as a timeline	Yes Figure4
Diagnostic Assessment	8a	Diagnostic testing (such as PE, laboratory testing, imaging, surveys).	Yes Figure1 AND 2 Lines 85—91
	8b	Diagnostic challenges (such as access to testing, financial, or cultural)	No
	8c	Diagnosis (including other diagnoses considered)	Yes
	8d	Prognosis (such as staging in oncology) where applicable	No, Not Applicable
Therapeutic Intervention	9a	Types of therapeutic intervention (such as pharmacologic, surgical, preventive, self-care)	Yes Lines 93—102
	9b	Administration of therapeutic intervention (such as dosage, strength, duration)	No, Not Applicable
	9c	Changes in therapeutic intervention (with rationale)	No
Follow-up and Outcomes	10a	Clinician and patient-assessed outcomes (if available)	Yes Lines 104—114
	10b	Important follow-up diagnostic and other test results	Yes Lines 104—114
	10c	Intervention adherence and tolerability (How was this assessed?)	No
	10d	Adverse and unanticipated events	No
Discussion	11a	A scientific discussion of the strengths AND limitations associated with this case report	Yes
	11b	Discussion of the relevant medical literature **with references**.	Yes
	11c	The scientific rationale for any conclusions (including assessment of possible causes)	Yes
	11d	The primary "take-away" lessons of this case report (without references) in a one paragraph conclusion	Yes
Patient Perspective	12	The patient should share their perspective in one to two paragraphs on the treatment(s) they received	Yes
Informed Consent	13	Did the patient give informed consent? Please provide if requested	Yes ☑ No ☐
